# The EU Rule of Law Initiative Towards the Western Balkans

**DOI:** 10.1007/s40803-020-00148-w

**Published:** 2020-11-18

**Authors:** Andi Hoxhaj

**Affiliations:** Warwick School of Law, University of Warwick, Coventry, UK

**Keywords:** Rule of law, Corruption, Organised crime, Enlargement policy, Open method of co-ordination, Western Balkans

## Abstract

The EU adopted a new enlargement strategy for the Western Balkans countries in 2018, provided a time frame for Serbia and Montenegro potentially to join the Union by 2025, and outlined the next steps for accession for Albania, Bosnia and Herzegovina, Kosovo, and North Macedonia. In March 2020, the EU gave the green light to the opening of accession talks with North Macedonia and Albania, and also introduced a new reformed ‘accession talks’ framework. The strengthening of the rule of law, fighting corruption and organised crime are the cornerstones of the EU-Western Balkans strategy of 2018 and the new accession talks framework of 2020. This article examines the latest enlargement policy developments in 2018–2020 by conceptualising how the EU promotes the rule of law in the Western Balkans thorough its new enlargement policy package. Furthermore, the article offers an in-depth analysis of the case of Albania, where the EU has experimented with some of its latest enlargement-policy ideas in regard to the rule of law. The article also offers some proposals and insights on how the EU rule of law initiative of 2018 can be improved, in order to become more transformative in strengthening the rule of law in countries of the Western Balkans.

## Introduction

The objective of this article is to explain the latest EU enlargement policy development in the years 2018–2020, and how the EU is trying to promote the importance of strengthening the rule of law in its latest enlargement policy package for six countries in the Western Balkans: Albania, Bosnia and Herzegovina, Kosovo, Montenegro, North Macedonia, and Serbia^1^. The contribution to scholarship made in this article aims to offer an analysis of the EU-Western Balkans strategy adopted in February 2018, and the newly revised ‘accession talks’ framework for Albania and North Macedonia adopted by the European Council in March 2020.

In particular, the focus of this article is to examine how the EU policy on the rule of law for candidate states has evolved over the years, and what the EU wants to achieve by reforming the judicial system (Appicciafuoco 2010). The article conceptualises how the EU is trying to promote the rule of law through its enlargement policy, offering an analysis of the ongoing judicial reform in Albania as a case study. The article also offers a discussion as to why the EU has made the reforming of the justice systems in the Western Balkans central to its new enlargement policy, in the light of the recent constitutional reforms in Poland and Hungary, which show signs of ‘democratic backsliding’ (Bátora and Fossum 2019) on the rule of law and liberal democratic standards. The thesis is that for the EU rule of law initiative to have a lasting impact in the Western Balkans, it is not sufficient to only focus on vetting the justice system, as in the case of Albania, to strengthen the rule of law.

The article also theorises the model of governance used by the European Commission in designing its rule of law initiative in the EU-Western Balkans Strategy of 2018, and explains its operation in practice. There is an analysis of the potential challenges that the rule of law initiative may face in becoming a transformative tool to strengthen the rule of law in the Western Balkans, given that there is a high preserved level of corruption, and furthermore, the argument that the legal and political conditions are not currently as favourable in the Western Balkans for the rule of law initiative to succeed, because these countries are semi-autocratic systems, and there is little political will to strengthen and subscribe to EU rule of law principles.

## The Latest EU Enlargement Policy Developments in 2018–2020

### The EU-Western Balkans Strategy of 2018

The EU’s enlargement policy on the Western Balkans has been running on ‘autopilot’ for the last fifteen years, and the accession process is perceived as a box-ticking exercise, both by scholars and by the European Parliament^2^. After the onset of the eurozone crisis at the end of 2009, EU enlargement no longer featured on the EU policy agenda (Gstohl 2016). However, after the Brexit vote, the Commission began to re-engage in its enlargement policy in the hope of keeping the EU project alive, and in the 2017 State of the Union address, the then Commission President Jean-Claude Juncker^3^ stated that the Western Balkans should become future members of the EU (Belloni and Brunazzo 2017). The EU Member States supported the Commission’s idea of extending the EU to the Western Balkans, and thus both the enlargement policy and the integration of the Western Balkans into the EU were restored and placed high on the EU policy agenda (Anghel 2018). Issues concerned with the rule of law, justice and fundamental rights (Berger 2019) were to be the central criteria in the EU accession talks with the Western Balkans, as emphasised in the 2017 State of the Union address^4^.

In February 2018, the Commission followed this up by adopting the ‘Credible Enlargement Perspective for an Enhanced EU Engagement with the Western Balkans’ strategy,^5^ which came almost fifteen years after the last EU-Western Balkans Summit, in Thessaloniki (Van Meurs 2003) in 2003^6^. The EU-Western Balkans strategy of 2018 introduced some renewed policy objectives on the future enlargement of the EU, and viewed the Western Balkans as a geostrategic investment for the Union bloc. At the same time, the EU also hoped to revive its future direction of Union in its entirety, after the damaging Brexit vote (Ker-Lindsay 2017). According to (Louwerse and Kassoti 2019), fulfilling the rule of law conditions set out in the Copenhagen criteria is key to the success of the Western Balkans in their accession talks with the EU.

The 2018 EU-Western Balkans strategy focuses on building good neighbourly relations, in light of the long-standing ethnic disputes in the region. Further, it sets out six ‘flagship initiatives’, which are areas of common interest to both the EU and the Western Balkans, namely: (1) the rule of law; (2) security and migration; (3) socio-economic development; (4) transport and energy connectivity; (5) the digital agenda; and (6) reconciliation and good neighbourly relations^7^. In essence, these flagship initiatives provide a framework and building blocks for developing future joint action plans in each of these six key areas, to be designed by the Commission and the respective Western Balkans governments, through a technical and political dialogue on reform aligned to EU governance standards (Wunsch, Kmezić, Stratulat and Tzifakis 2019). In this article, I consider and conceptualise the rule of law initiative, accompanied by a theoretical discussion of how the EU has identified these flagship initiatives as key priority areas for reform during the accession negotiations phase, and the design of the governance model for their practical operation—which at first glance appears to be based on the Open Method of Coordination.

In 2019, a year after the EU-Western Balkans strategy, the EU published country progress reports^8^ for each of the Western Balkans countries. The reports provided an in-depth assessment of the progress made by each of the Western Balkans countries in fulfilling the EU accession criteria^9^—in particular, progress towards meeting the criteria related to the rule of law (Sargentini and Dimitrovs 2016). On this occasion, the Commission also made proposals to the 27 EU Member States for its Council meeting to discuss the state of affairs in the Western Balkans, especially considering whether these countries were complying with the Copenhagen criteria related to the function of the rule of law (Fouéré 2019). The main proposals issued to the 27 EU Member States were that the Commission identified Serbia and Montenegro as front-runners for EU accession, and recommended that both countries be allowed to join the Union by 2025^10^. Whilst this theoretical date was intended to encourage both Serbia and Montenegro to continue their internal reforms towards fulfilling the EU *acquis*,^11^ a sustainable track record and tangible progress towards the Copenhagen criteria^12^ (especially Chapter 23 and 24 of the *acquis,* on ‘judiciary and fundamental rights’^13^ and ‘security and justice’^14^ (Hillion 2016) were clearly identified as being indispensable, in order to have any hope of joining the EU in such a timeframe. For Albania^15^ and North Macedonia,^16^ the Commission proposed that the Member States grant the opening of accession talks with no further pre-conditions. Albania had to pass a comprehensive judicial reform with a view to strengthening the rule of law and making progress in complying with the Copenhagen criteria, which was adopted in June 2016, by changing more than one-third of its constitution as a major pre-condition to the opening of accession talks with the EU (Hoxhaj 2020)^17^. North Macedonia, in contrast, as a major pre-condition, had to resolve a three-decade-long dispute about its name with its neighbour Greece (Bechev 2019)^18^. In 2018, both countries signed the Treaty of Prespa, which included changing the name of the country from the ‘Former Yugoslav Republic of Macedonia’ to the ‘Republic of North Macedonia’ (Vankovska 2020). The Commission proposed to the Member States that Bosnia and Herzegovina^19^ be granted the EU candidate status, and that visa liberalisation be granted to Kosovo^20^.

In the conclusions of the June 2019 Council meeting, the 27 EU Member States acknowledged the progress achieved in the Western Balkans based upon the Commission’s findings^21^. However, Member States continued to express concern regarding the Western Balkans’ compliance with the Copenhagen criteria and the lack of the rule of law, and thus did not endorse the country report recommendations to open accession talks with Albania and North Macedonia and grant visa liberalisation to Kosovo, and they made no commitment to granting EU candidate status to Bosnia and Herzegovina—although the Member States suggested that they would review the Commission proposal to start accession talks with Albania and North Macedonia in the autumn of 2019. Moreover, the Member States indicated that they viewed accession in 2025 as creating an extremely ambitious timetable for Serbia and Montenegro to join the Union. Notwithstanding the reservations and hesitation from Members States on specific steps forward in expanding the EU in the Western Balkans, there was a general consensus to support the EU foreign and security policy objective as a long-term geopolitical strategy and security investment (Burlyuk 2020).

### The Rule of Law Flagship Initiative in the EU-Western Balkans Strategy of 2018

In the 2018 EU-Western Balkans strategy it is acknowledged that there are clear elements of state capture, including links with organised crime and corruption at all levels of government and administration in the Western Balkans, and the rule of law is weak (Richter and Wunsch 2019). Thus, the rule of law initiative ought to be one of the most important initiatives of the six flagship initiatives in the strategy, and should receive more attention from both the EU and the Western Balkans if they are to crack down on state capture and seriously fight corruption at the highest level (Elbasani and Šabić 2018).

When evaluating the EU-Western Balkans strategy of 2018, there are as yet no concrete proposals nor a roadmap for strengthening the rule of law, but only some broad policy objectives. We should rather understand the strategy as a platform for the Western Balkans and the Commission to engage in a dialogue to transform the rule of law flagship initiative into a joint action plan. Reforming the judicial system is an end goal, a by-product of which should serve to strengthen independent institutions to be able to uphold the rule of law without political interference^22^. The strategy indicates that the Commission will work closely with the Western Balkans towards ensuring that the judiciary is reformed in line with the highest EU standards and the Copenhagen criteria, and will offer technical and financial support in the fight against corruption and organised crime as part of the rule of law initiative. There are no clear examples of the EU and the Western Balkans developing a joint action plan or strategy on fighting corruption and organised crime, or how such cooperation would fit within the overall initiative on rule of law as yet, since there is no joint action plan as a direct outcome of the rule of law initiative, nor explicit indication of what any help might include—but it seems that it may involve expanding the operation of Frontex (the European Border and Coast Guard Agency) to the Western Balkans, with a view to fighting transnational organised-crime networks that use a route through the Western Balkans to access the EU^23^.

The implicit principle in the rule of law initiative of the 2018 EU-Western Balkans strategy is that a clear track record in the fight against corruption and organised crime at the highest level is required, in order to show true commitment towards meeting the core EU membership criteria (Müller 2015) regarding both the rule of law and the overall Copenhagen criteria, if countries want to be taken seriously in their bids to join the EU (Hillion 2011). In other words, the Western Balkans must show a record of indictments of public officials who have either abused their power or engaged in corruption, or been part of or associated with an organised-crime network, as the first step of good faith in their commitments towards the Copenhagen criteria. This is a clear indication that the EU will not take the Western Balkans seriously if there is a continuation of political interference (Fagan and Kopecký 2018) in judicial decisions, investigations, and indictments of high-level officials. Furthermore, the underlying message in the rule of law initiative is that the Commission plans to make use of all of the leverage provided in the accession talks frameworks for as long as possible, by delaying the Western Balkans accession to the EU in order to avoid any repetition of the scenarios of Hungary and Poland (Grabbe and Lehne 2017), where there were clear elements of backsliding in their commitments to the rule of law (Adamski 2019), or, in the cases of Bulgaria,^24^ Slovakia,^25^ and Malta,^26^ where high-profile politicians were observably involved in corruption and organised-crime networks.^27^

However, further analysis is required with regard to the proposal to develop a joint ‘action plan to reform the judiciary’, under the rule of law initiative in the EU-Western Balkans strategy of 2018—which the Commission, together with Western Balkans countries, may develop in the next two years.^28^ To date, no action plan has been published as a direct outcome of the rule of law initiative, and it is difficult to assess how it will be operated in practice once deployed. However, there are some signs that the operation and governance model of the rule of law initiative is based on the Open Method of Coordination (OMC). The next section, I discuss the theory of the model of governance that the Commission used, and how it may work in practice when fully developed. Furthermore, the theory behind the OMC can help in understanding the operation of rule of law initiative for the Western Balkans.

### The Rule of Law Initiative as a Form of the Open Method of Coordination

In analysing the rule of law initiative, one can see that the main outcome appears to aim at producing a joint action plan on how the Western Balkans can reform its judicial systems, with an end goal of sustainable improvement in the rule of law, to be acceptable to EU liberal democratic standards and in line with the Copenhagen criteria. One could argue that the framework for how the Commission aims to work with the Western Balkans governments in producing the joint action plan is largely based upon the OMC model. Let us now briefly examine this model, considering its impact on the design of the rule of law initiative for the Western Balkans, and the potential for further use of the OMC model once the joint action plans are deployed in practice.

The OMC (Zeitlin 2005) is an instrument of European integration, and its method is applied in policy fields where the main competences still rest with the Member States. It is a soft law instrument which employs non-binding objectives and guidelines to bring about change in law and policy areas. The term ‘OMC’ was introduced in the 2000 Lisbon Strategy (Dawson 2011) to achieve greater convergence between Member States in areas where the EU did not have competence—most notably, labour market and social policies (Rogowski 2015). Erika Szyszczak (2006) argues that the advent of the OMC in EU policymaking and governance models builds upon the long tradition of soft-law processes used in policymaking, and in experimentation with new forms of governance, drawing upon the success of the Commission’s monitoring of traditional hard law directives and the peer review ‘name and shame’ mechanisms, utilised in the implementation and monitoring of the internal market programme, which have gradually been extended to other policy areas (De la Porte and Pochet 2012); this now also appears in the EU enlargement policy.

It must be noted that there are differences between the various OMC models, depending on the policy area in question, but usually the procedures and overall format is the same (Dawson 2011). First, the Member States in the Council meetings set policy goals with the recommendations of the Commission, which are then applied in each of the Member States according to their needs. Second, implementation is evaluated against benchmarks and indicators which have been agreed upon among the participants in the process—usually, there is time period of two years, and successive steps are laid out through an action plan which establishes when the policy goals or reforms should take place. Third, the results of the implementation are evaluated by the Commission, and also compared against best practices between each of the Member States, in order to identify which country might be the best example to follow in the next cycle (Rogowski 2015). The end results of the evaluations by the Commission and the recommendations for the next two-year cycle are not explicitly binding for Member States, because the process itself is not legally binding. However, the ‘peer pressure’ element in the OMC model usually encourages Member States to be more proactive in implementing deeper reforms than they might otherwise have done without such external scrutiny, as they now run the risk of being compared unfavourably to their fellow Member States (Beveridge and Velluti 2016).

These elements and aspects of the OMC model can be found throughout the EU-Western Balkans strategy of 2018—in particular, with regard to how the rule of law initiative is to be deployed in practice. Once the joint action plan has been developed and the rule of law initiative is implemented, it will include establishing guidelines, quantitative and qualitative indicators and benchmarks, and national and regional targets backed by periodic evaluations and peer review, possibly every two years. The Commission indicates that the key objective of the joint action plans is the reform and transformation of the judicial system—in other words, it aims to ensure the safeguarding of the rule of law against the benchmark set in the Copenhagen criteria (Mendelski 2016a). This explicit approach, coupled with the use of developing impact indicators, trial-monitoring, and case-based peer-review missions to monitor the implementation of the judicial reform in the joint action plan, are indicators that the OMC is the Commission’s model of governance for its enlargement policy, promoting the strengthening of the rule of law by reforming the judiciary (Kmezić 2017).

Therefore, when evaluating the EU-Western Balkans Six Flagship Initiative, one could argue that the Commission has envisioned its implementation based upon the OMC model. The Commission suggests that the rule of law initiative aims to foster mutual learning (Armstrong and Kilpatrick 2007) about successful judicial reforms between the Western Balkans countries, and learn best practices from Member States which had weak rule of law architecture when they initially joined the EU—such as Bulgaria, Croatia, and Romania (Mungiu-Pippidi 2018). In summary, the rule of law initiative in the EU-Western Balkans 2018 strategy seems to have been designed by the Commission as a soft law instrument, relying on the OMC model as one proven to have been successfully used by the Commission in other policy areas, and which is now being experimented with in EU enlargement policy, with a focus on reforming the judicial system as a means to strengthen the rule of law in the Western Balkans.

At the time of writing (September 2020), no action plan has been developed as a direct outcome of the rule of law initiative. Analysing an existing plan would have made it easier to establish the extent to which the Commission in the 2018 EU-Western Balkans strategy has based its application of the rule of law initiative on the OMC model in practical terms. For the present, the elements of the OMC are only visible in the design of the rule of law initiative—but future research may contribute to analysing the governance model in greater depth, once the action plan has been developed and applied in practice.

The postponement of the joint action plan and the implementation of the six flagship initiatives of the EU-Western Balkans strategy of 2018 is mainly attributed to the reluctance on the part of some Member States to accept the current design of the EU enlargement policy (Mendelski 2016b)—in particular, the accession talks framework. In March 2020, at the request of France, the Commission came up with a new accession talks framework for the Western Balkans. It is not yet clear how the rule of law initiative proposed in the 2018 EU-Western Balkans strategy will be applied in the light of this new accession talks framework, differing as it does from the previous one, in which the six flagship initiatives were tailored. However, it might include of a mixture of hard and soft law instruments. This approach diverges from the OMC model of governance, as some Member States want to have more direct involvement in the EU enlargement process in the future, rather than leave it to the Commission. This shift was recently made more concrete, as the next section will explain in more depth. Issues concerning fundamental rights, and in particular the rule of law, will be central to the new accession talks framework for the Western Balkans.

## The New 2020 Accession Talks Framework for Albania and North Macedonia

North Macedonia has been a candidate for EU membership since 2005, and Albania since 2014. Over the past few years, the Commission has championed the idea that both North Macedonia and Albania are ready to open accession talks with the Union. However, Member States have not agreed to this—and in October 2019 the situation came to a head, when France, the Netherlands and Denmark strongly opposed opening accession talks with the two countries, in particular Albania.

In the October 2019 Council meeting, France blocked the opening of accession talks with North Macedonia and Albania by presenting two main arguments for their veto: first, the EU needs to strengthen its existing policies and institutions before adding any new members; and second, that the enlargement policy and accession talks process is flawed, as there is no guarantee that the candidate state will subscribe to the Copenhagen criteria and uphold the rule of law and the EU liberal democratic values once they join the EU. The main rationale behind France’s decision was based upon the notion that, once a country becomes a Member State of the EU, there are no adequate mechanisms to address subsequent backsliding of democratic standards and the rule of law (Smith 2019); these concerns come in light of the ongoing developments in Hungary and Poland. These are valid arguments when one considers the backsliding of liberal democratic standards in some Member States, and it must conceded that the EU requires reform in order to bolster the eurozone, and its decision-making processes (Rhinard 2019) in responding more swiftly to crises—highlighted even more by the COVID-19 pandemic crises.

However, France was heavily criticised both by its fellow Member States and the Commission, for damaging the credibility of the EU as a geopolitical power in the Western Balkans through its veto to open accession talks with Albania and North Macedonia^29^. Furthermore, Member States criticised the French government decision to block accession talks because they were not based upon reforming the EU enlargement policy, but were more a response to internal French politics in order to bolster President Macron’s stand against the Eurosceptics in France who openly oppose European enlargement. The same conclusion can be drawn for other Member States where Eurosceptic parties are on the rise, and there is an increase in the opposition to further European integration (Kuhn 2019). One should also not underestimate the rise of Islamophobia within the internal politics of some Member States (Jackson 2018), given that Albania is a country with a large Muslim population, and that 25 per cent of the population of North Macedonia are ethnic Albanians who share the Muslim faith. There are Member States uncomfortable with the idea of a country with a large Muslim majority^30^ ever joining the EU (Noutcheva and Düzgit 2011). However, this is not a determining factor at the moment, since all of the six Western Balkans countries together comprise about 18 million people, and Albania’s population is only about 3.4 million, of which almost 1.4 million are already living abroad. Currently the main debate about the EU enlargement towards the Western Balkans is more focused on issues concerning widespread corruption, organised crime and state capture, and the restricted capacity for independent institutions to uphold the rule of law.

In its response to the criticism described above, France published a proposal on how to reform the EU accession process framework, entitled the ‘French Non-paper: Reversibility Needed in New Enlargement Strategy’,^31^ published in November 2019. This later become an influential proposal on how the Commission could move forward in its engagement with the Western Balkans and put the rule of law as the central criteria for upcoming negotiation talks with Albania and North Macedonia. The French proposal suggested that the future EU accession process should not be based upon opening and closing the 35 chapters of the *acquis*, but should be transformed into a process of seven phases, concerning: (1) the rule of law and fundamental rights; (2) education and research; (3) employment and social affairs; (4) financial affairs; (5) the single market, agriculture, and fisheries; (6) foreign affairs; and (7) ‘others’ In other words, under this proposed format, the 35 chapters of *acquis* are to be restructured into these seven phases, which would then form policy blocs. The issues concerning the rule of law and fundamental rights (Huszka and Körtvélyesi 2017), were proposed first, and were therefore central to the new accession talks framework—and an indication that Member States will pay greater attention to the rule of law in candidate countries in the future.

France suggested that once a country opened accession talks, it would start going through the first phase, and the EU would reward each successfully completed stage with entry to different EU structures and programmes (such as Eurojust, the research and science programme, the EU banking union, and perhaps access to the EU’s customs union and the single market or Europol)^32^. The rationale of the ‘rewards’ is that, in the previous format of ‘opening and closing the 35 chapters of the *acquis*’, it was difficult to explain—not only to the citizens of the candidate country, but also to the voters in the 27 EU Member States—what were the associated benefits^33^. The French proposal also suggested that the Member States should have a more direct say in the accession talks, and have the option of cancelling the talks if they see a candidate country backsliding on the rule of law or other fundamental values of the EU^34^. This approach represents a drastic shift from the previous policy, where Member States left the technical side of the accession talks up to the Commission. Member States having the option to cancel accession talks due to backsliding goes beyond the soft law mechanism used by the Commission, modelled around the OMC, and is instead a hard law approach based on a ‘sticks and carrots’ strategy to promote the rule of law (Himmrich 2019).

In January 2020, when the new Commission’s new President Ursula von der Leyen took office, the Commission was tasked to revise the accession talks framework using the French proposal as a basis; reforming the rule of law was central to the thinking on how to improve the framework. On 5 February 2020, the Commission published its own proposal for reforming the framework, entitled ‘New Methodology for the Accession Negotiations’,^35^ adopted by the Member States in the Council in March 2020. The main changes according the ‘New Methodology’ document is that the EU candidate country will no longer negotiate its accession into the EU based upon the 35 chapters of *acquis* framework, as is currently being used for Serbia, and Montenegro, and these 35 chapters are to be reorganised into six thematic clusters: (1) Fundamentals; (2) Internal Market; (3) Competitiveness and Inclusive Growth; (4) Green Agenda and Sustainable Connectivity; (5) Resources, Agriculture, and Cohesion; and (6) External Relations. The first cluster, Fundamentals, is focused mainly on respecting and upholding EU fundamental rights, and on reforming the independent institutions such as the judicial system, so that the state is able to uphold the rule of law and democratic standards laid out in the Copenhagen criteria.

This new accession talks framework of 2020 has incorporated the major recommendations proposed by France. The Commission, in line with the French proposal, has suggested more tangible rewards to EU candidate states—such as integrating them into certain EU policy areas, possibly allowing them to enter into the banking union, the educational programme, and participating in the European arrest warrant scheme, and also injecting more direct funding and investment to boost their economies. However, such rewards are only on offer to candidate states able to show tangible progress and a track record in upholding the rule of law.

On 24 March 2020, the foreign ministers of the 27 Member States reached a political consensus to give the green light^36^ to start accession talks with North Macedonia and Albania, based upon the new accession talks framework of 2020^37^. The Member States were satisfied with the Commission recommendations to open accession talks with North Macedonia and no further conditions were deemed necessary. However, for Albania, the Member States rejected the Commission recommendations to open accession talks unconditionally^38^. Instead, the Member States attached a set of 15 pre-conditions which must be fulfilled before Albania is technically permitted to open accession talks. Thus, the case of Albania is more complex, and not based on a soft law mechanism alone, but a mix of hard and soft law that goes beyond the OMC model upon which the Commission seems to have originally based its policy for strengthening the rule of law in the Western Balkans.

## The 15 Pre-accession Conditions for Albania

First, Albania must go through a two-stage process for the 15 pre-accession conditions which must be fulfilled by the first and second accession conferences,^39^ which will involve officials from the EU and Albania^40^. This is the first time that such a format has been applied for any candidate state before it can technically open accession talks with the Union. These conditions were largely based upon a resolution that the German Parliament (Rüttershoff 2020) adopted in September 2019, when it gave the mandate to the German government to give a pro-vote in the Council meeting in October 2019 to open accession talks with Albania, subject to its meeting nine pre-accession conditions. In addition to the nine conditions established by the *Bundestag*, another six pre-accession conditions were also added to accommodate the concerns of France, Greece, Denmark, and the Netherlands, regarding Albania’s accession to the EU.

*By the first Accession Conference, Albania has to fulfil six pre-conditions*:

- The reforming and passing of the Albanian electoral law—in line with the recommendations of the OSCE Office for Democratic Institutions and Human Rights (ODIHR), and the adoption of more transparency rules for political campaigns and party funding;
- The further implementation of judicial reform in line with the opinions of the Venice Commission^41^: in particular, the Constitutional Court and the Supreme Court must reconvene after being suspended since judicial reform was adopted in 2016;
- The Albania Anti-Corruption Prosecutor’s Office (SPAK) should start its work with indictments, and a National Bureau of Investigation (NBI) should be established^42^;
- The establishing of a track record in the fight against corruption, organised crime, and money laundering;
- The controlling and sanctioning of the phenomenon of unfounded asylum applications to the Member States—rejected asylum seekers must be returned to Albania promptly; and
- The safeguarding of the freedom of the press and the amendment of the media law—in particular, the so-called ‘anti-defamation package’—in line with the opinions of the Venice Commission.

*After Albania has satisfactorily fulfilled these six pre-conditions, it then must meet an additional nine pre-conditions before the second Accession Conference takes place*:

- Criminal proceedings and indictments must be issued for judges and prosecutors who failed the re-evaluation ‘vetting’ process;
- Criminal investigations should be opened for those accused of buying votes in the 2017 parliamentary elections and the 2019 local elections. Election fraud instruments are to be further strengthened for the upcoming parliamentary election set for June 2021;
- A track record in fighting corruption and organised crime should be established, showing a clear record for proceedings against high-ranking public officials;
- Further progress must be exhibited in reforming the public administration;
- The implementation of the new electoral law for the upcoming 2021 parliamentary election must be enacted;
- The Constitutional Court should issue a final verdict on the legality and validity of the local elections that took place in June 2019;
- The further implementation of the 2017 legislation on the protection of national minorities;
- A new census law that is in line with the recommendations of the Council of Europe must be adopted; and
- The law on property rights and ownership must be amended, and the capacities for the registration of property should be increased.

Incorporating and attaching these fifteen pre-accession conditions was the only way to reach a political consensus in the Council meeting (Rüttershoff 2020) between the rest of the Member States and the more reluctant members, Denmark, France, Germany, Greece, and the Netherlands, in order to obtain their political approval to open accession talks with Albania. At a glance, one may imagine that some of these pre-conditions for Albania will likely require a longer period of time to be fulfilled, and, more importantly, pose difficulties for establishing evidence quantifiable as a ‘satisfactory’ track record—in the eyes of the more reluctant Member States—especially, in the fight against corruption and organised crime. One of the pre-accession conditions, for example, requires ‘investigating and prosecuting corruption and organised crime at the highest level of public office’. Even if there is a strong political will on the part of Albania to achieve this, quick results cannot be demonstrated, due to the complexity that such investigations require. Furthermore, the language used in the pre-accession conditions is vague, and can be open to interpretation as to how one might measure or quantify whether the progress made by Albania is acceptable for some of the more sceptical Member States; *i.e.*, how many politicians, public officials, judges, and prosecutors should be imprisoned in order to obtain a ‘satisfactory track record’?

Let us consider Romania as an example, whose National Anti-corruption Directorate the EU has promoted as a success model for the Western Balkans to follow, where conviction rates are about 90 per cent; the country has, to date, indicted over 4,700 defendants since the Directorate was established in 2002 (Hoxhaj 2020). This includes jailing a prime minister, 18 ministers, and thousands of mid-level public officials and politicians on charges of corruption and abuse of public office. It must be noted that Romania’s National Anti-corruption Directorate’s successful track record of indictments and convictions only began in 2010 (Mungiu-Pippidi 2018)—eight years after its initiation. The Albanian Anti-Corruption Prosecutor’s Office (SPAK) was only established in late 2019, and one cannot anticipate that Albania could have a similar successful track record such as that seen in Romania until at least 2030, especially in indicting high-ranking profile public officials.

Nevertheless, there are some encouraging signs, such as SPAK charging the former Attorney General of Albania Adriatik Llalla (who served from 2012 to 2017) with failure to declare the full extent of his property—this case is pending in the courts^43^. It must be noted that there was huge pressure to investigate the former Attorney General due to the US State Department, under section 7031(c) of the FY 2017 Consolidated Appropriations Act,^44^ publicly barring Llalla and his family from entering the United States due to ‘his involvement in significant corruption’ in February 2018^45^. Two other discredited Albanian politicians have been publicly barred by the government from US entry in 2018^46^ and 2019,^47^ and privately, it is believed that hundreds of politicians, judges, and prosecutors are ineligible for entry to the US under suspicion of being involved in corruption and organised crime.

In Albania’s case, the EU has tried, as part of its enlargement policy efforts, to support the fight against corruption and organised crime by promoting judicial system reform as the first major step in strengthening the rule of law, in line with the Copenhagen criteria. However, in so doing, the EU has not used a soft law approach such as described in the OMC, but more of a traditional hard law approach. The section below explains how the EU has made the judicial reform a pre-requisite in Albania. Given that all of the Western Balkans countries have serious issues with high levels of corruption and close links between high-ranking public officials and organised crime networks, what follows is an insight into how the EU’s Albanian judicial reform model—as the first major step in strengthening the rule of law—might be replicated across the rest of the Western Balkans.

## The EU Rule of Law Initiative and Policy Experimentation in Albania

The judicial system in Albania is perceived as being highly corrupt, with very close links to politicians and organised-crime networks^48^. As a result, the EU has identified the judicial system as one of the most corrupt public sectors in every annual progress report since Albania was granted the EU candidate status in 2014. In its EU conditionalities, the Commission has repeatedly suggested that if Albania doesn’t reform the judicial system in line with the Copenhagen criteria, it is unlikely that accession talks will be open between Albania and the EU (Vurmo 2020). As a result of the EU conditionalities and internal political pressure to make progress in opening accession talks with the EU, the Albanian Parliament appointed a special committee in November 2014, with a mandate to make proposals for the reform of the justice system in line with the EU conditionalities and the Copenhagen criteria. The committee had three tasks: (1) to provide an in-depth analysis of the function of the judiciary; (2) to outline the main objectives of the judicial reform; and (3) to propose amendments to the laws that will require changes in order to enable the implementation of the judicial reforms, especially the constitutional amendments in line with the EU conditionalities, which would allow Albania—on paper, at least—to open and close chapters of the *acquis* chapters 23 and 24, related to the rule of law and fundamental rights (Hoxhaj 2020).

The parliamentary committee was assisted by a technical secretariat, and in particular by international experts, which ensured that Albania was developing its proposal for judicial reform in line with the EU conditionalities. The two main international experts are the EU’s ‘Consolidation of the Justice System in Albania’ project (EURALIUS),^49^ and the US Department of Justice’s Overseas Prosecutorial Development, Assistance, and Training Program (known as OPDAT),^50^ both of which offered technical assistance in drafting the judicial reform. These experts are currently monitoring the judicial reform implementation process, and are expected to continue to do so for a full eight years. In September 2015, the group of experts presented the draft legal package of the judicial reform, which the Albanian parliamentary committee forwarded to the Council of Europe’s Venice Commission for its opinions (Anastasi 2018) to certify its constitutionality.

The Venice Commission issued its response in early 2016, and made recommendations on how judicial reform might be implemented and monitored by an international monitoring body composed of former judges and prosecutors from the EU and the US, which would offer insurance that the reform was going to be implemented in line with the Copenhagen criteria. It also made recommendations on the constitutional changes to enable the first part of implementing the judicial reform legal package, and how the powers of the new anti-corruption bodies should be constrained under the constitution (Richard, Benvindo, Rado and Zhilla 2017). Almost all the political parties accepted the Venice Commission’s recommendations in principle, but there was disagreement between members of the governing Socialist Party of Albania and the Democratic Party of Albania opposition party about a legal clause which would have allowed EU and US magistrates to monitor the implementation of the judicial reform—in particular, during the vetting process of the judges and prosecutors in Albania. The approach proposed by the EU is a sign that the Member States have deep distrust in candidate states’ ability to reform themselves. It also suggests that judicial reform in Albania is based on a hard law mechanism, rather than the soft law found in the EU approach of the OMC model. Ultimately, under huge pressure from the Commission and leaders of EU government, and even more so from the US government, all 140 MPs in the Albanian parliament (Gjevori 2018) bowed to external pressure, and voted unanimously in favour of the constitutional changes in June 2016, in order to open the path to the first phase of implementing the judicial reform, which—in theory—would put Albania in a favourable position to open accession talks with the EU.

The legal package for judicial reform included the introduction of 46 new constitution articles, which were all related to reorganising the judicial system. The constitutional changes would also restructure the relationship between the executive and the legislative branches, with the judicial branch being allowed more autonomy. The main changes to the Constitution can be summarised as follows (Hoxhaj 2020):

- The establishing of new vetting bodies, entitled the ‘International Monitoring Operation’^51^ and the ‘Independent Committee of Qualifications’,^52^ with a legal mandate to re-evaluate all of the 800 members of the judiciary;
- The establishing of two new investigative bodies to fight corruption and organised crime, entitled the ‘Special Anti-Corruption and Organised Crime Structure’, and the ‘National Bureau of Investigation’;
- The Constitutional Court and the Supreme Court should be transformed into career courts, and nominations and appointments of their members should not be carried out by the executive branch, to reduce the potential for political influence;
- Judges and prosecutors are to be given more autonomy, and to be selected through a self-governing body that can propose and appoint future members to serve in the judiciary, including the selection and nomination of the attorney general.

The implementation of judicial reform started in late 2017, with a single focus on vetting all 800 officials of the Albanian judiciary. The vetting of these officials is based upon three criteria: (1) assets; (2) background; and (3) proficiency assessment (Hoxhaj 2020). To date, the results of the vetting process on almost half of the officials has been dismissal, or in some cases voluntary resignation, because of failure to justify their assets.

The Constitutional Court and the Supreme Court were given priority when the vetting process began, and, after the re-evaluation of the judges sitting in the Constitutional Court, seven out of the nine judges proved inadequate to occupy a post in the judiciary (Bara and Bara 2017). Of the Supreme Court judges, only four of nineteen passed the vetting process. Judges in both the Constitutional and Supreme Courts were unable to justify their assets. The indication is that they failed the vetting process because their assets had been acquired through bribery or corruption related to the cases that they had adjudicated (Hoxhaj 2020).

Corruption in the judicial system, and more broadly in other spheres of public life is widespread in Albania, as well as in the rest of the Western Balkans (Zvekic 2017). According to the Balkan Barometer of 2018, citizens of the Western Balkans are placing corruption as the third ‘burning issue’ in the region, after unemployment and healthcare.^53^ Similar findings were identified by the latest Transparency International Corruption Index (CPI) and the Commission progress reports for the Western Balkans in 2019. As the section below indicates, the level of corruption in the countries of the Western Balkans is higher than most EU Member States; only Bulgaria scored the same as the Western Balkans.

For the Commission’s rule of law flagship initiative to be successful when fully developed and deployed in practice, consideration must be given to how challenging it will be to successfully strengthen the rule of law, as the conditions are so unfavourable in the Western Balkans. First, there is a high level of corruption, and second, most of the Western Balkans states are semi-autocratic or hybrid democracies (Bieber 2018), with little political will to fulfil the EU rule of law conditionalities, and, just as importantly, to uphold them.

### The Level of Corruption in the Western Balkans Weakens the Rule of Law

The Transparency International Index (CPI) of 2019 indicates that corruption levels in the Western Balkans are stagnating. Apart from Montenegro, each country in the region has achieved between 36 and 39 points, which puts them between 87 and 99 places out of 180 countries in the CPI^54^. Kosovo, Montenegro and Serbia saw perceived corruption levels rise in the past year, while Bosnia and Herzegovina recorded its worst result since 2012. North Macedonia and Albania share the worst ranking in the Western Balkans. Notwithstanding the CPI’s limitations, country scores give a useful snapshot for researchers and policymakers about the level of corruption in the Western Balkans, and the possible challenges for the rule of law initiative to be a transformative tool in strengthening the rule of law in this region.

The CPI 2019 found that Serbia scored 39, dropping two points since last year^55^. This trend can be explained as the Serbian government continues to undermine the institutions that are responsible for maintaining the rule of law. In 2018, despite strong opposition from NGOs, lawyers’ associations and other civil society activists, the government pushed for increased influence over the judiciary^56^. Furthermore, the government has openly violated and amended its own anti-corruption policies, proposed as a response to the 2013–2018 Anti-Corruption Strategy and Action Plan for Chapter 23 EU Integration (2016–2018)^57^.

Bosnia and Herzegovina scored 36—the country’s worst ranking since 2012, thereby dropping 11 places compared to 2018 in the CPI^58^. According to the report by Transparency International, the deterioration of the level of corruption in Bosnia and Herzegovina is linked to the irregularities of election campaigns, new laws related to financing political parties and voting suppression. In other words, in Bosnia and Herzegovina there is an increase of political corruption, and—even though there is evidence of election fraud according to the findings of Transparency International—judicial institutions have so far been unable to take action to uphold the rule of law^59^.

Kosovo witnesses a similar trend in corruption, dropping further in 2019, only scoring 36 in the CPI^60^. There were high hopes in early 2020, when the Transparency International report suggested that Kosovo is ‘a country to watch’, due to a change of government led by the Vetevendosje party, who came into power with a promise to clean up corrupt public institutions and reform the judiciary to strengthen overall the rule of law^61^. However, this government only lasted for 50 days, and in that time did not implement any such reforms.

Montenegro scored 45 in the CPI in 2019, better than the rest of the Western Balkans states^62^. However, corruption and state capture remain problematic, as the government in 2019 proposed amendments to the ‘law on classified information’. According to Transparency International and a coalition of 25 Montenegrin NGOs,^63^ the amendments proposed by the government could undermine the country’s freedom of information laws, the anti-corruption efforts, and overall the function of the rule of law, because the proposed law allows the government to declare information classified at its discretion. In its progress report in 2019, the Commission^64^ suggested that Montenegro should reverse the growing trend of public institutions declaring information classified as a matter of priority, as this prevents any oversight by independent institutions or civil society on issues related to corruption, and therefore, limits the ability of the judicial system to uphold the rule of law.

North Macedonia shared the worst result in the Western Balkans together with Albania, scoring just 35 points in the CPI in 2019^65^. The Transparency Report and the Commission country report^66^ suggest that North Macedonia has made good progress in the fight against corruption, and further consolidating a track record of prosecuting and adjudicating high-level corruption cases. However, in 2018–19, the country’s rule of law was tested to its core, after its special prosecutor for organised crime and corruption Katica Janeva^67^ was arrested on suspicion of offering leniency in exchange for a bribe to a businessman indicted for corruption. In June 2020, Janeva was sentenced to seven years’ imprisonment after being found guilty of accepting bribes and luxury gifts as part of an extortion scheme^68^. Janeva’s case has fuelled major political turmoil in North Macedonia over the last two years, and has been a major test for the courts to demonstrate their ability to uphold the rule of law.

In 2019, *Albania* dropped seven places in the CPI, scoring 35 points out of 100, and has dropped 23 places in just over three years since the judicial reform begun^69^. As a result, Albania is perceived to be the most corrupt country in the Western Balkans. Both the Freedom House report for Albania and Transparency International found that issues of conflict of interest, abuse of state resources for personal and electoral purposes, insufficient disclosure of political party and campaign financing, and a lack of media independence are prevalent in the Albania^70^. Despite a number of anti-corruption reforms since the judicial reform was adopted, corruption is widespread across the state, and is impeding its ability to uphold the rule of law (Hoxhaj 2020).

The levels of corruption indicated by the Transparency International 2019 CPI in all Western Balkans states is clearly impeding its chances to make a successful case for EU integration. In these conditions, where corruption its high and justice can be bought and sold through bribery, the overall ability of law enforcement and the judicial system to uphold the rule of law is profoundly undermined. However, corruption is only one of the negative factors challenging the rule of law initiative in the Western Balkans. Crucially, there is also very little political will—both to fight this corruption and to strengthen the rule of law overall—in semi-authoritarian political systems (Kmezić 2020).

### Strengthening the Rule of Law in Autocracies

The governments in the Western Balkans have been described as ‘stabilocracies’ by (Bieber 2020a, b), and the leaders of the government can be understood as autocrats that capture the state, and claim to secure stability in the Western Balkans region by pretending to champion European integration. However, these governments leaders rely on informal, clientelist structures, controlling the media, and regularly producing artificial political crises over EU conditionalities to undermine any true efforts in strengthening the rule of law (Radeljić and Đorđević 2020).

In the majority of the Western Balkan countries, autocrats are in power, and thus only a handful of people control the economy and the distribution of political authority (Bieber 2020b). Checks and balances, and the separation of powers between the judicial, executive and legislative branch are weak almost to the point of inexistence. Therefore, government leaders have almost absolute control over the country’s affairs^71^—and in this environment, the space to strengthen the rule of law, or expose organised-crime networks and corruption becomes far more challenging. So far in the Western Balkans, political elites often simply avoid prosecution, as was the case with the former Prime Minister of North Macedonia, Nikola Gruevski, who fled to Hungary^72^ days before he was due to be jailed on charges of abuse of power and corruption.

Despite EU conditions on curbing corruption and ensuring institutions (particularly the judicial system) are free of political influence, many governments across the Western Balkans have so far failed to show any true political will to strengthen the rule of law (Kmezić 2020). Political elites have excessive and unchecked power, and there are limited mechanisms to hold them to account. Strengthening the rule of law (Mendelski 2018) is clearly in opposition to the interests of those autocrats in power, and thus, far less likely to succeed. Perhaps this is why, since 2018, there has been no action plan to strengthen the rule of law in the Western Balkans as a direct outcome of the EU-Western Balkans strategy?

In an environment where public institutions cannot fully safeguard the rule of law, due to state capture by autocratic leaders, it is imperative that civil society actors, such as journalist, academics and NGOs are supported to play a more central role in scrutinising (Stojarová 2020) the reforms related to EU rule of law conditionalities. So far, the theoretical design of the rule of law initiative is largely based on the OMC model, which entitles a discussion between members of the Commission and the governments on the rule of law reform—but there is no formal framework for the inclusion of civil society actors. However, the Commission may use this opportunity to include civil society in the implementation phase of the rule of law initiative, and more importantly in the design of national action plans related to the strengthening of the rule of law in each of the Western Balkans countries—such inclusion could be a significant factor in the initiative becoming a transformative tool.

## Lessons Learned From Albania and Improving the Rule of Law Initiative

The first step in the EU-Western Balkans 2018 Enlargement Strategy Rule of Law Initiative is judicial reform. As shown in Albania, this approach mainly involves vetting the members of judiciary. However, this primary focus must expand to make space for civil society actors to participate, moving away from the top-down approach which includes only EU and state government officials. Furthermore, the rule of law initiative must also include new investment in the judicial system’s infrastructure and management (Becker 2018), making it more accessible for citizens. Vetting judicial officials is a crucial part of the rule of law initiative, but in moving forward, it is just as important that the judiciary is funded appropriately—and here the rule of law initiative may play a role in coordinating investments in the Western Balkans justice system.

Compared to the average across the 27 EU Member States, the judiciary systems in the Western Balkans are underfunded, and allocated budgets are mostly for day-to-day running costs. The Council of Europe ‘Plan of Action to Strengthen the Judicial Independence and Impartiality’^73^ suggests that budgetary constraint (Kocevska 2019) is a way to implicitly control the resource mobilisation process of the judiciary system. In other words, judicial independence is difficult to achieve without financial freedom.

The European Commission for the Efficiency of Justice (CEPEJ),^74^ in its latest report published in 2018, suggests that the judiciary in the Western Balkans does indeed face budgetary constraint in comparison to EU Member States. Montenegro and Bosnia and Herzegovina distributed 1–1.4% of GDP to the judicial system; Albania, North Macedonia and Serbia judicial system budgets are between 0.26–0.8% of GDP. This means that the judicial system budget per capita in the Western Balkans is highest in Montenegro and Bosnia and Herzegovina, both allocating around 25 euros per capita, whereas North Macedonia allocates 20 euros, and Albania allocates less than 10 euros per inhabitant for the judicial system. The average EU Member State judicial system budget allocation is 64 euros per capita, with a median value of 53 euros per capita^75^.

This data suggests that the Western Balkans countries are not allocating sufficient funding to the judiciary in comparison to the EU member states and thus, when the joint action plan is developed, there must be an increase in such funding. If commensurate funding were to be allocated in Albania, perhaps—as of September 2020—the country would not have over 35,000 cases pending adjudication? Since the start of the justice reform, the country has been without a Constitutional and Supreme Court, and critics suggest that this unprecedented situation has opened the door for constitutional violation by the government, in itself a threat to the rule of law. Both courts are anticipated to start functioning by the end of 2020 or early 2021, but as long as Albania continues to be without a functional Constitutional and Supreme Court, the government has the ability to freely pass legislation in parliament without due care to the constitutionality of laws, as there is no court with the legal mechanism in place to oversee whether legislation may infringe upon the constitution and the rule of law.

The fact of the large number of cases pending in Albanian courts has received major criticism with regard to the judicial reform the EU has promoted, and critics suggest that as long as the courts are not working, this represents a violation of the Constitution and the European Convention on Human Rights (Gerards and Glas 2017), as citizens have not had appropriate access to justice system since the judicial reform was passed in June 2016^76^. The USAID and the Albanian High Judicial Council signed a Memorandum of Understanding (MOU) in April 2020 as a result of this criticism, to provide some financial and technical assistance in order to deal with the workload accumulated as a result of the courts not working^77^. Notwithstanding this attempt to offset some of the damage, this is a lesson that the EU may learn from Albania—and the rule of law initiative could prioritise the allocation of appropriate resources and funding in the rest of the Western Balkans, to prevent significant impairment of the judicial system whilst vetting is ongoing.

What the Albania example shows is that the EU enlargement policy has made the issue regarding the rule of law central to its newly revised ‘Enlargement Policy And Accession Talks Framework’ in EU candidate states. Reforming the judiciary is a major part of the conditionalities of the EU enlargement policy post-2018. The requirement is to strengthen the rule of law, thus not only meeting the Copenhagen criteria on paper, but demonstrating a tangible track record of subscribing to the EU model of liberal democracy.

It is too early to draw a concrete conclusion whether the Albania rule of law reform is a success story as a result of the EU rule of law conditionalities until the whole process has been completed, and it’s still too soon to decide if the EU model used in Albania can be championed in the rest of the Western Balkans through the rule of law initiative—and yet, so far, the process is delivering some encouraging results, as half of the vetted judicial officials have been expelled from the judicial system due to unexplained assets and wealth acquired through corruption.

However, what Albania has clearly already demonstrated is that the EU cannot rely too heavily on soft law mechanisms and those based on the OMC model of governance, as designed so far in the EU-Western Balkans 2018 strategy. The OMC model of governance is an important soft law mechanism that can help both the Commission and members of the Western Balkans governments to achieve the technical standard part of the rule of law reform. In particular, the OMC could ensure that the quality of the legal framework (as observed in the case of Albania) is aligned with the Copenhagen criteria, before constitutional changes can be made to allow the implementation of rule of law-related reform later. Furthermore, the OMC model of governance used by the Commission can help the Western Balkans to learn best practice from the EU in improving the legal framework, once it has been initially drafted. However, in order to acquire the broad political consensus and the political will—by all political parties—to actually adopt judicial reform and make the necessary constitutional changes that can allow the implementation of rule of law-related reforms, a soft law instrument such as the OMC model may be insufficient. This is because major disagreements can occur— for example, in Albania, with regard to the authorising of EU and US former magistrates to monitor and empower the vetting and potential dismissal of members of the judiciary in a candidate state. In practical and political terms, such imposition is an unpalatable approach in the current climate—not only in the Western Balkans but in the EU Member States—as it appears that national sovereignty is being disrupted when executive power over judicial matters is shifted to an international body or EU-affiliated bodies. In Albania, getting judicial reform through parliament, and making the necessary amendments to the constitution was only possible via huge external pressure from influential EU governments such as Germany, and in particular through US government influence over Albania politicians (Mustafaj 2020). Internal political processes, even when framed as responding to meeting EU conditionalities and therefore giving the country a better chance of opening accession talks with the EU, were not enough to initiate or sustain such important judicial reform (Mustafaj 2020) that might strengthen overall the rule of law.

## Conclusion

The latest EU enlargement policy developments are an indication that, post-Brexit, the Union wants to revive its enlargement project with future expansion into the Western Balkans. By targeting areas of common interest, the six flagship initiatives in the EU-Western Balkans 2018 strategy can be seen as proof of the EU’s renewed policy efforts to support the Western Balkans to resolve and make sustainable progress in their internal socio-economic issues. The initiative to strengthen the rule of law in the EU-Western Balkans 2018 strategy, if applied in an orderly manner, may accelerate the process of making the judicial systems of the Western Balkans less prone to corruption, with a clear system of check and balance, a more independent judicial system from the executive branch, and more closely aligned with the EU best practice standards.

However, strengthening the rule of law on paper, by passing a judicial reform package as in the case of Albania, shows that it is not sufficient to strengthen and uphold the rule of law in practice. The rule of law initiative for the Western Balkans is a promising new approach by the European Commission, and, with the introduction of the new accession talks framework for Albania and North Macedonia in 2020, it has the potential to generate a more productive platform for the EU and the Western Balkans to engage in an evidence-based policy dialogue. However, the EU policy objective should not only be focused on vetting judicial officials as observed in Albania. Policy should also focus on institutional capacity-building, and investment in the judicial infrastructure so that it is more accessible to the citizens of the Western Balkans.

The rule of law initiative so far has only served as a platform for dialogue between the Commission and members of the Western Balkans, and although it based on a soft law mechanism after the OMC model of governance, it is very much a top-down approach, and has not fully engaged with members of the civil society. In the absence of independent institutions and state capture, formally including members of the civil society in designing national action plans on reforming the rule of law in the Western Balkans can help both the EU and the Western Balkans to ensure that governments are implementing the rule of law-related reforms in line with the EU conditionalities, as well as empowering civil society to take a more proactive role in upholding the rule of law.

## Footnotes

1 Albania, Montenegro, North Macedonia, and Serbia and are official candidate countries for accession to the European Union. Bosnia and Herzegovina, and Kosovo are potential candidates for the EU.

2 European Parliament (2015) The Western Balkans and EU Enlargement: Lessons learned, ways forward and prospects ahead. Policy Department, Directorate-General for External Policies. (online) Available at: https://www.europarl.europa.eu/RegData/etudes/IDAN/2015/534999/EXPO_IDA(2015)534999_EN.pdf Accessed 20 May 2020.

3 European Commission President Jean-Claude Juncker, State of the Union Address, 13 September 2017: ‘If we want more stability in our neighbourhood, then we must maintain a credible enlargement perspective for the Western Balkans. It is clear that there will be no further enlargement during the mandate of this Commission and this Parliament. No candidate is ready. But thereafter the European Union will be greater than 27 in number. Accession candidates must give the rule of law, justice and fundamental rights utmost priority in the negotiations.’ (online) Available at: https://ec.europa.eu/commission/presscorner/detail/en/fs_19_6414 Accessed 12 August 2020.

4 European Commission President Jean-Claude Juncker, State of the Union Address, 13 September 2017. (online) Available at: https://ec.europa.eu/commission/presscorner/detail/en/fs_19_6414 Accessed 12 August 2020.

5 European Commission (2018), ‘Communication on a Credible Enlargement Perspective for and Enhanced EU Engagement with the Western Balkans’, COM (2018) 65 final.

6 In the EU-Western Balkans Summit in 2003 in Thessaloniki, the EU initially made a pledge to expand the Union into the Western Balkans. See the European Commission Press Release (18 June 2003), ‘The Thessaloniki Summit: A Milestone in the European Union's Relations with the Western Balkans’. (online) Available at: https://ec.europa.eu/commission/presscorner/detail/en/IP_03_860 Accessed 20 May 2020.

7 European Commission (2018), ‘Communication on A credible enlargement perspective for and enhanced EU engagement with the Western Balkans’, COM (2018) 65 final.

8 In the country reports, the Commission usually makes recommendations and introduces the conditions that each Candidate State must meet before it can make further progress towards joining the EU—it mainly includes adopting laws and policies in areas related to the 35 chapters of the *acquis* ‘accession chapters or criteria’.

9 The Treaty on the European Union sets out the conditions (Article 49) and principles (Article 6(1)) to which any country wishing to become an EU member must conform. These criteria are known as the Copenhagen criteria, and were established by the Copenhagen European Council in 1993 and later strengthened by the Madrid European Council in 1995. For EU accession negotiations to be launched, a country must satisfy the first criteria, and they are as following: (1) stability of institutions guaranteeing democracy, the rule of law, human rights and respect for and protection of minorities; (2) a functioning market economy and the ability to cope with competitive pressure and market forces within the EU; (3) ability to take on the obligations of membership, including the capacity to effectively implement the rules, standards and policies that make up the body of EU law (the 'acquis'), and adherence to the aims of political, economic and monetary union. (online) Available at: https://eur-lex.europa.eu/summary/glossary/accession_criteria_copenhague.html Accessed 12 August 2020.

10 European Commission (2018), ‘Communication on a Credible Enlargement Perspective for an Enhanced EU Engagement with the Western Balkans’, COM (2018) 65 final.

11 The *Community acquis* or *acquis Communautaire*, sometimes also called the *EU acquis* and often shortened to *acquis*, is the accumulated legislation, legal acts, and court decisions which constitute the body of European Union law. During the process of the enlargement of the European Union, the *acquis* is divided into 35 chapters for the purpose of negotiation between the EU and the Candidate Member States.

12 Since the Copenhagen European Council in 1993, the EU has defined that the candidate country has to achieve stability of institutions guaranteeing democracy, the rule of law, and human rights (known as the Copenhagen criteria). These key elements later became Chapters 23 and 24, and fundamental to the accession process.

13 The elements compiled under Chapter 23 are closely linked to the political criteria which need to be met for overall negotiations to begin. They include four main headings: the judiciary; the fight against corruption; fundamental rights; and EU citizens’ rights.

14 Chapter 24 covers the fight against all types of organised crime, including drug and arms trafficking, the trafficking in human beings, and terrorism. It also includes alignment with the Schengen rules, border control and visas, as well as migration, asylum, judicial co-operation in criminal and civil matters, and police and customs cooperation.

15 European Commission (2019), ‘Communication from the Commission to the European Parliament, the Council, the European Economic and Social Committee and the Committee of the Regions: 2019 Communication on EU Enlargement Policy on Albania 2019 Report’ COM (2019) 260 final.

16 ibid.

17 Hoxhaj 2020, p.168.

18 Bechev 2019, p.26–28.

19 European Commission (2019), ‘Communication from the Commission to the European Parliament and the Council Commission Opinion on Bosnia and Herzegovina’s application for membership of the European Union’, COM (2019) 261 final.

20 European Commission (2019) ‘Communication from the Commission to the European Parliament, the Council, the European Economic and Social Committee and the Committee of the Regions: 2019 Communication on EU Enlargement Policy on Albania 2019 Report’ COM (2019) 260 final.

21 European Council (2019), ‘Council Conclusions on Enlargement and Stabilisation and Association Process’ Press release. (online) Available at: https://www.consilium.europa.eu/en/press/press-releases/2019/06/18/council-conclusions-on-enlargement-and-stabilisation-and-association-process Accessed 20 May 2020.

22 European Commission (2018), ‘Communication on a Credible Enlargement Perspective for an Enhanced EU Engagement with the Western Balkans’, COM (2018) 65 final.

23 In 2019, Frontex fledged a joint operation outside the EU for the first time by deploying over 50 officers to Albania in supporting border control, with a view to fighting translational crime networks that use the Western Balkans route through Albania to smuggle migrants and illegal narcotics and migration into the Union. See Frontex News Release (2019) Frontex Management Board Meets with Partners from the Western Balkans. (online) Available at: https://frontex.europa.eu/media-centre/news-release/frontex-management-board-meets-with-partners-from-the-western-balkans-1wf8k8 Accessed 20 May 2020.

24 In Bulgaria, an investigative journalist, Viktoria Marinova, was killed in 2018 for investigationg an alleged fraud involving EU funds linked to businessmen and politicians. (online) Available at: https://www.theguardian.com/world/2018/oct/07/tv-journalist-brutally-murdered-in-bulgarian-town-of-ruse Accessed 20 May 2020.

25 In Slovakia, an investigative journalist, Ján Kuciak, was killed in 2018 for discovering high levels of corruption and links to organised crime. (online) Available at: https://www.dw.com/en/in-memory-of-ján-kuciak-the-immortal-search-for-truth/a-52464911 Accessed 20 May 2020.

26 In Malta, an investigative journalist, Daphne Caruana Galizia, was killed in 2017 for undercovering politicians linked to corruption and organised crime. In 2019, the Malta’s PM, Joseph Muscat, resigned as a result of the investigation into the murder of the journalist. (online) Available at: https://www.theguardian.com/membership/2020/feb/08/malta-daphne-caruana-galizia-murder-journalist-investigation Accessed 20 May 2020.

27 European Parliament (2019) Press Release on Malta and Slovakia: Serious Shortcomings in the Rule of Law. (online) Available at https://www.europarl.europa.eu/news/en/press-room/20190218IPR26964/malta-and-slovakia-serious-shortcomings-in-the-rule-of-law Accessed 20 May 2020.

28 European Commission (2018), ‘Communication on a Credible Enlargement Perspective for an Enhanced EU Engagement with the Western Balkans’, COM (2018) 65 final.

29 Financial Time Editorial Board (2019), ‘Emmanuel Macron’s EU Accession Veto is a Historic Mistake: Bloc should Open Talks with North Macedonia and Albania, then Reform itself’ (online). Available at: https://www.ft.com/content/eda39e1e-f3eb-11e9-b018-3ef8794b17c6 Accessed 20 May 2020.

30 It is well documented that in early 2000, when Turkey was actively more pro-European and its foreign policy goal was to join the EU, there were reservations on the part of some Member States about allowing a country with a large Muslim population to be part of the Union. EU Members have been mulling over either suspending or ending the talks since November 2016, as the Turkish government policies under Erdogan are viewed by the majority of the Member States to have eroded the rule of law and human rights standards.

31 ‘Reforming the European Union Accession Process’, Non-Paper, November 2019 (online) Available at: https://www.politico.eu/wp-content/uploads/2019/11/Enlargement-nonpaper.pdf Accessed 20 May 2020.

32 The EU enlargement process under French proposal would be more gradual, and once a EU Candidate State graduates from the seventh phase, it could become a full member of the European Union.

33 Unlike visa liberalisation, where citizens of the Western Balkans could perceive the progress more directly related to them, and see the positive difference of not having to obtain a visa in order to enter the EU Schengen Area.

34 The Member States are concerned that the Western Balkans might become the next Hungary or Poland within the EU, given that the majority of the countries in the Western Balkans are led by autocrats with little respect for the rule of law, human rights, and/or liberal democratic values.

35 European Commission (2020), ‘Communication on Enhancing the Accession Process: A Credible EU Perspective for the Western Balkans’, Communication from The Commission to The European Parliament, The Council, The European Economic and Social Committee and The Committee of The Regions, COM (2020) 57 final.

36 European Commission (2020) Press release: ‘Commission Welcomes the Green Light to Opening of Accession Talks with Albania and North Macedonia’ (online) Available at: https://ec.europa.eu/neighbourhood-enlargement/news_corner/news/commission-welcomes-green-light-opening-accession-talks-albania-and-north-macedonia_en Accessed 20 May 2020.

37 European Commission (2020) Press release: ‘Commission Reports on Progress Made by Albania and North Macedonia’ (online) Available at: https://ec.europa.eu/commission/presscorner/detail/en/IP_20_347 Accessed 20 May 2020.

38 European Council (2020) Press release: ‘Council Conclusions on Enlargement and Stabilisation and Association Process—Albania and the Republic of North Macedonia’ (online) Available at: https://www.consilium.europa.eu/en/press/press-releases/2020/03/25/council-conclusions-on-enlargement-and-stabilisation-and-association-process Accessed 20 May 2020.

39 The accession conference is a setting where the EU and Albania will discuss the 15 pre-accession conditions in depth, and Albania must show credible progress in fulfilling these pre-accession conditions in the two accession conferences. Only after Albania has shown credible evidence in meeting the 15 pre-accession conditions in the two accession conferences can the EU accession talks formally begun.

40 After Albania has fulfilled the 15 pre-accession conditions, the EU and Albania will technically open accession talks in an intergovernmental conference. The Commission will provide a timetable for the first accession conference, after the Covid-19 crisis, and has postponed a potential date until late autumn of 2020. (online) Available at: https://www.euractiv.com/section/enlargement/news/enlargement-package-postponed-until-autumn-negotiation-framework-to-go-ahead-in-june Accessed 20 May 2020.

41 The Venice Commission, in a draft opinion issued on 29 May 2020, criticised a number of decisions and procedures followed in the vetting process. In particular, the Commission stressed that Albania has had a dysfunctional Constitutional Court and High Court for more than three years, and it must re-start working as soon as possible. It also made a recommendation that the vetting commissions term of nine years is ‘excessive’, because the vetting process needed to be ‘strictly temporary’. (online) Available at: https://exit.al/wp-content/uploads/2020/05/CDL2020016-e-1.pdf (Accessed: 31 May 2020).

42 In Albania this is known as ‘SPAK’: the special anti-corruption and anti-organized crime proxy is a new constitutional institution, which will have the task of investigating and prosecuting corruption and organised. SPAK is composed of two organs: the special prosecutor’s office (composed of prosecutors), and the national investigative agency (composed of investigators). The national investigative agency is the operational arm of SPAK, modelled on the American FBI, who have also financed it. SPAK only started to function in September of 2019.

43 The United States Department Communication on Public Designation of Adriatik Llalla under section 7031(c) of the FY 2017 Consolidated Appropriations Act. (online) Available at: https://www.state.gov/public-designation-of-adriatik-llalla-under-section-7031c-of-the-fy-2017-consolidated-appropriations-act Accessed 20 May 2020.

44 The US Consolidated Appropriations Act 2017 builds on the Magnitsky Act of 2012, which authorises the US government to sanction those whom it sees as human rights offenders, freezing their assets and banning them from entering the US.

45 The US Consolidated Appropriations Act 2017, in cases where it has credible information that foreign officials have been involved in significant corruption or violations of human rights, those individuals and their immediate family members are ineligible for entry into the US.

46 The United States Department Communication on Public Designation of Tom Doshi under Sect. 7031(c) of the FY 2017 Consolidated Appropriations Act. (online) Available at: https://www.state.gov/public-designation-of-tom-doshi-under-section-7031c-of-the-fy-2017-consolidated-appropriations-act Accessed 20 May 2020.

47 The United States Department Communication on Public Designation Due to Involvement in Significant Corruption of Albanian Mayor of Durrës, Vangjush Dako. (online) Available at: https://www.state.gov/public-designation-due-to-involvement-in-significant-corruption-of-albanian-mayor-of-durres-vangjush-dako Accessed 20 May 2020.

48 Albania’s Vetting Commission dismissed in 2018 a ‘High Court Judge’ over finding connections with organised crime groups. (online) Available at: https://balkaninsight.com/2018/07/30/albania-high-court-judge-vetted-over-connection-with-organized-crime-07-30-2018 Accessed 20 May 2020.

49 EURALIUS is an EU-funded technical assistance project that seeks the strengthening of the Albanian Justice. (online) Available at: https://euralius.eu/index.php/en Accessed 20 May 2020.

50 OPDAT in Albania is involved in reviewing criminal-justice legislation; training judges, police and prosecutors; providing law-enforcement equipment; and providing technical assistance aimed at establishing more effective law enforcement structures. (online) Available at: https://www.justice.gov/criminal-opdat Accessed 20 May 2020.

51 The International Monitoring Operation is a monitoring operation set up to oversee the vetting process of judiciary members in Albania. (online) Available at: https://www.cilc.nl/project/international-monitoring-operation (Accessed: 20 May 2020).

52 The Independent Qualification Commission is a constitutional entity directly responsible for the assessment and re-evaluation of judges, prosecutors, inspectors, legal advisors and other members of Albania's justice system. It has revised some criticism in 2020 regards to the transparency of re-evaluating judicial officials, as some judges and prosecutors that have engaged in petty corruption or served during Communism have passed the vetting process—it was hard to see how they passed the vetting process against the integrity criteria. See for more: Vladimir Karaj (2020) ‘Transparency Questions over Albanian Effort to Rid Courts of Corruption’ *Balkan Insight*. (online) Available at: https://balkaninsight.com/2020/01/24/transparency-questions-over-albanian-effort-to-rid-courts-of-corruption (Accessed: 20 May 2020).

53 Regional Cooperation Council, ‘Balkan Barometer 2018: Public Opinion Survey’ 2018, https://www.rcc.int/pubs/66/balkan-barometer-2018-public-opinion-survey .

54 Maja Zivanovic, (2020) ‘New Transparency Report Gives Balkan Countries Poor Scores’. Balkan Insight. (online) Available at: https://balkaninsight.com/2019/01/29/balkan-countries-perceived-as-corrupted-report-01-28-2019/ Accessed 5 September 2020.

55 Transparency International (2020) ‘Serbia 2019 CPI’ (online) Available at: https://www.transparency.org/en/countries/serbia Accessed 5 September 2020.

56 Martin Russell, (2019) ‘Serbia at risk of authoritarianism? European Parliamentary Research Service’ (online) Available at: https://www.europarl.europa.eu/RegData/etudes/BRIE/2019/637944/EPRS_BRI(2019)637944_EN.pdf?fbclid=IwAR1irVOQ0Lyrixf8HNnute555Y79WRg8yFBWaz6OarDkOfPW3FBeXJB_PWs Accessed 5 September 2020.

57 European Commission, (2019) ‘Communication from the Commission to the European Parliament, the Council, the European Economic and Social Committee and the Committee of the Regions 2019 Communication on EU Enlargement Policy – Serbia 2019 Report’. (COM(2019) 260 final) (online):ilable at https://ec.europa.eu/neighbourhood-enlargement/sites/near/files/20190529-serbia-report.pdf Accessed 5 September 2020.

58 The Transparency International (2020) ‘Bosnia and Herzegovina 2019 CPI’ (online) Available at: https://www.transparency.org/en/countries/bosnia-and-herzegovina Accessed 5 September 2020.

59 EU Special Representative in Bosnia and Herzegovina, (2019) ‘Expert Report on Rule of Law issues in Bosnia and Herzegovina’ (online) Available at: https://europa.ba/wp-content/uploads/2019/12/ExpertReportonRuleofLawissuesinBosniaandHerzegovina.pdf?fbclid=IwAR0ihgFYkVjq76Sv0Fr9lyncKA56WtPIFXjUZzE9jPwmsD40lxHNJCogFEo Accessed 5 September 2020.

60 The Transparency International (2020) ‘Kosovo 2019 CPI’. (online) Available at: https://www.transparency.org/en/countries/kosovo Accessed 5 September 2020.

61 Aidan Hehir, (2020) ‘Kosovo’s government just fell – but it’s down to US meddling rather than coronavirus. The Coversation’ (online) Available at: https://theconversation.com/kosovos-government-just-fell-but-its-down-to-us-meddling-rather-than-coronavirus-134862 Accessed 5 September 2020.

62 The Transparency International (2020) ‘Montenegro 2019 CPI’ (online) Available at: https://www.transparency.org/en/countries/montenegro Accessed 5 September 2020.

63 The Transparency International, (2019) ‘Montenegro – Widely condemnded law does not deserve a second chance’ (online) Available at: https://www.transparency.org/en/press/montenegro-widely-condemnded-law-does-not-deserve-a-second-chance Accessed 5 September 2020.

64 European Commission, (2019) ‘Communication from the Commission to the European Parliament, the Council, the European Economic and Social Committee and the Committee of the Regions 2019 Communication on EU Enlargement Policy – Montenegro 2019 Report’. (COM(2019) 260 final) (online) Available at: https://ec.europa.eu/neighbourhood-enlargement/sites/near/files/20190529-montenegro-report.pdf Accessed 5 September 2020.

65 The Transparency International, (2020) ‘North Macedonia 2019 CPI’ (online) Available at: https://www.transparency.org/en/countries/macedonia-fyr Accessed 5 September 2020.

66 European Commission, (2020) ‘Update on the Republic of North Macedonia’. SWD (2020) 47 final (online) Available at: https://ec.europa.eu/neighbourhood-enlargement/sites/near/files/update-on-the-republic-of-north-macedonia.pdf Accessed 5 September 2020.

67 Sinisa Jakov Marusic, (2019) ‘North Macedonia Arrests Outgoing Chief Special Prosecutor’. Balkan Insight. (online) Available at: https://balkaninsight.com/2019/08/21/north-macedonia-arrests-outgoing-chief-special-prosecutor/ Accessed 5 September 2020.

68 Sinisa Jakov Marusic, (2020) ‘North Macedonia Jails Ex-Special Prosecutor Over “Extortion” Case’. Balkan Insight (online) Available at: https://balkaninsight.com/2020/06/18/north-macedonia-jails-ex-special-prosecutor-over-extortion-case/ Accessed 5 September 2020.

69 The Transparency International, (2020) ‘Albania 2019 CPI’ (online) Available at: https://www.transparency.org/en/countries/albania Accessed 5 September 2020.

70 Freedom House, (2020) ‘Nation in Transition: Albania 2020 country report’ (online) Available at: https://freedomhouse.org/country/albania/nations-transit/2020 Accessed 5 September 2020.

71 Jovan Bliznakovski, Borjan Gjuzelov and Misha Popovikj, (2017) ‘The Informal Life of Political Parties in the Western Balkan Societies’. University College London, INFORMAL project (online) Available at: https://www.ucl.ac.uk/ssees/sites/ssees/files/idscs-informal_life_of_political_parties-report-27092017.pdf

72 Sinisa Jakov Marusic, (2019) ‘Hungary Refuses to Extradite Gruevski to North Macedonia’. Balkan Insight (online) Available at: https://balkaninsight.com/2019/06/27/hungary-refuses-to-extradite-gruevski-to-north-macedonia/ Accessed 5 September 2020.

73 Council of Europe, (2016) ‘Plan of Action to Strengthen the Judicial Independence and Impartiality’. CM (2016)36 final (online) Available at: https://www.coe.int/en/web/cdcj/judicial-independence-and-impartiality Accessed 5 September 2020.

74 The CEPEJ was established in 2002 by the Committee of Ministers of Council of Europe, and publishes a report every two years assessing the efficiency of judicial systems in the 47 Council of Europe Member States.

75 European Judicial Systems Efficiency and Quality of Justice, 2018, p.26.

76 It can be argued the judicial reform has technically violated Articles 6 and 13 of the European Convention on Human Rights, as citizens have not been able to have access to justice since 2016, even though the Albanian government and the EU has justified the suspension of the courts as necessary to root out the level of corruption, and allow time for the vetting process. (online) Available at: https://europeanwesternbalkans.com/2019/11/13/vetting-process-in-albania-the-marching-failure Accessed 20 May 2020.

77 Euronews Albania, (2020) ‘USAID to Assist Albanian Courts with 35,000 Pending Cases’ (online) Available at: https://euronews.al/en/albania/2020/04/30/usaid-to-assist-albanian-courts-with-35-000-cases-backlog Accessed 20 May 2020.
